# Helping the waiter to hold his tray: Rigid haptic linkage promotes inter-personal motor coordination

**DOI:** 10.1177/17470218211009082

**Published:** 2021-04-12

**Authors:** Dardo N Ferreiro, Chris D Frith, Bahador Bahrami

**Affiliations:** 1 Department of General Psychology and Education, Ludwig-Maximilian-University Munich, Munich, Germany; 2 Department Biology II, Division of Neurobiology, Ludwig-Maximilian-University Munich, Germany; 3 Wellcome Centre for Human Neuroimaging, University College London, London, UK; 4 Institute of Philosophy, School of Advanced Study, University of London, London, UK; 5 Centre for Adaptive Rationality, Max Planck Institute for Human Development, Berlin, Germany; 6 Department of Psychology, Royal Holloway, University of London, London, UK

**Keywords:** Interpersonal coordination, sensorimotor, forward model, haptics, collaboration

## Abstract

When a glass is lifted from a tray, there is a challenge for the waiter. He must quickly compensate for the reduction in the weight of the tray to keep it balanced. This compensation is easily achieved if the waiter lifts the glass himself. Because he has, himself, initiated the action, he can predict the timing and the magnitude of the perturbation of the tray and respond (via the holding hand) accordingly. In this study, we examined coordination when either one or two people hold the tray while either one of them or a third person removes the glass. Our results show that there is exquisite coordination between the two people holding the tray. We suggest that this coordination depends upon the haptic link provided by the rigid platform that both people are holding. We conclude that the guest at a reception should not lift his drink from the waiter’s tray until they have the waiter’s attention but, if too thirsty to wait, should lend a hand holding the tray.

## Introduction

The thirsty guest at reception should not lift his drink from the waiter’s tray. He should wait for the waiter to hand it to him. When the glass is lifted from the tray, there is a challenge for the waiter. He must quickly compensate for the reduction in the weight of the tray (i.e., generate a downwards movement) to keep the tray balanced. This compensation is much more easily achieved if the waiter lifts the glass himself. Because he has, himself, initiated the action of lifting the glass off the tray, he can predict the timing and perturbation of the tray and prepare the response of the hand holding the tray. According to predictive-control theory, the waiter will achieve this by using a forward-model, that is, an internal model that can be used to predict the consequences of his action (Hugon et al., 1982; Miall & Wolpert, 1996; Pezzulo & Cisek, 2016). This has two measurable consequences. First, the compensatory response to the removal of the glass from the tray will be initiated earlier and, second, the amplitude of the perturbation will be smaller. This is an example of bi-manual coordination between the hand doing the lifting and the hand holding the tray. This coordination critically depends on the availability of a signal enabling the holding hand to take compensatory action at the appropriate time. There is evidence that this signal has to come from within the motor system directly concerned with the lifting action. Presenting a tone that signals the onset of lifting does not reduce the perturbation. Similarly, the perturbation is not reduced when the participant, rather than performing the lifting action, initiates the lift by pressing a button (Dufossé et al., 1985).

In this study, we explored bi-manual coordination in the case where the two hands belong to different people. Pezzulo and colleagues (2017) have previously explored the possibility that two people could coordinate their tray holding behaviour by having them both simultaneously lift a glass from the other’s tray. The idea is that each person can use their expectation from their own action to predict the effect of the other person’s action. This hypothesis was confirmed: simultaneous lifting (bimanual coordination) was associated with significantly less perturbation of the tray.

In our study, rather than simultaneous lifting from different trays, we explored the coordination achieved when the tray is held by two hands. We would expect good coordination when the tray is held by one person using both hands, but how well can two people coordinate when they hold the tray with one hand each? They can, of course, see each other, but, in addition, they are connected by the rigid tray that they are both holding. In other words, they are linked haptically. Haptic linkage of this kind has been shown to facilitate coordination (Reed et al., 2006; van der Wel et al., 2011). Two people holding the tray together may simply *move less*, rigidly holding the tray in the same place and being less perturbed when a third person lifts the glass. Alternatively, cooperating teams may be able to coordinate better—that is to say, more precisely and with less perturbation.

We examined coordination when either one or two people were holding the tray while a third person removed a glass and also when one of them lifted the glass. We measured two aspects of the perturbation: (1) the variance in the vertical tray movement around the time that the glass was lifted and (2) the time course of the tray-holder’s response to the lifting of the glass. We expected that there would be more stability during the compensation of the perturbation when the tray was held with two hands rather than one (Hypothesis 1). Extending the predictive control theory to interpersonal coordination, we examined whether this reduction was as great when the two hands belonged to different people (Hypothesis 2). We also predicted that the response onset to the perturbation would be earlier when the glass was lifted by someone holding the tray (Hypothesis 3). Through use of the forward model, this person would be able to more precisely predict the moment that the glass would start rising. Finally, we examined whether the response would be equally early when the tray was being held by two people, only one of whom was lifting the glass (Hypothesis 4). Our results suggest that there is, indeed, exquisite coordination between the two people holding the tray.

## Methods

Participants (122 total, 67 female, 55 male, mean age = 35.7 ± 15.8 years) were visitors at “Tate Exchange” at Tate Modern in May 2019. Given that the experiment was conducted at a public event rather than in laboratory conditions, we were able to analyse data from 70 participants (see Data Analysis for details). Upon entering the clearly marked testing area at the museum’s fifth floor of the Blavatnik Building, participants gave their informed consent that their anonymized data could be used for research purposes. The experimental procedure was approved by the University of London Ethics (ASREC_1819-313) and approved by the organising team at the Tate museum. As the experiment was conducted as part of a public event, we did not control for participants knowing each other. Most people who participated together in pairs knew each other beforehand; all others were randomly matched with a partner. As is customary in public science events, we welcomed volunteers irrespective of their age meaning that several children and adolescents (accompanied by their parents) also participated in our experiment. These data, however, were not used in the study.

## Experimental conditions and procedure

**Figure 1.**
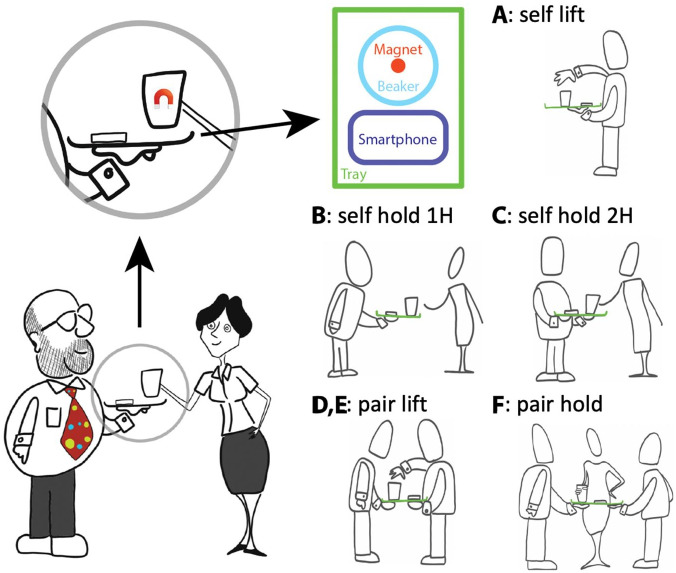
Experimental paradigm. Left: general schematic of the task. One or two participants (here one with a colourful tie) held a tray while the experimenter (or participant) removed the beaker from the tray. The accelerometer and magnetometer signals from the smartphone were recorded. Magnetometer signal was used to infer the timing of the beaker’s movement. Accelerometer signal was used to infer the tray motion. Right: Experimental Conditions A through F.

Participants were asked to hold a tray with their left hand, while either themselves or the experimenter removed the beaker with their right hand. The tray containing a smartphone and a beaker (Figure 1). A small magnet was fixed inside the beaker. The smartphone was fixed to the tray using Velcro tape. The arrangement of the tray, smartphone, and beaker was used to measure two variables: (1) the acceleration of the tray movement and (2) the displacement of the beaker (relative to the tray) as it was removed.

Using in-house developed custom software for Android (see Supplementary Material for open source code and tutorial), we recorded the perturbations of the smartphone’s accelerometer (for later analysis of tray movement) and magnetometer (for later analysis of the beaker movement relative to the tray) with a sampling rate of ~ 60 Hz.

Six experimental conditions (illustrated in Figure 1), three individual (A, B, and C) and three paired (D, E, and F) were tested:

A (self-lift): Participant held the tray with the left hand (whole hand under tray) and lifted the beaker with the right hand.
B (self-hold 1 H): Participant held the tray with the left hand, experimenter lifted the beaker with the right hand.
C (self-hold 2 H): Participant held the tray with both hands, experimenter lifted the beaker with the right hand.
D (pair-lift): Two participants held the tray with their left hands (whole hands under tray); one participant (P1) lifted the beaker with the right hand.
E (pair-lift): Same as D, only this time P2 lifted the beaker.
*F* (pair-hold): Two participants held the tray with their left hands together, experimenter lifted the beaker with the right hand.

Experimental conditions (individual or paired), as well as the order in which they were performed, were pseudo randomly assigned to participants. Each participant in the individual condition performed three repetitions of A, B, and C; each pair of participants (each dyad) performed three repetitions of D, E, and F. Therefore, each participant (or dyad) performed one recording consisting of nine consecutive trials (i.e., recordings of individual participant include combinations of trials such as CCCAAABBB, and recordings of pairs include combinations such as FFFEEEDDD, see Online Supplementary Figure 1 for examples of recordings). Data were obtained for 88 recordings.

### Data analysis

All data were processed and analysed with custom-written codes in MATLAB. Of the 88 recordings obtained, 38 were discarded before analysis. 10 were discarded due to participants being underage, and the rest either due to inadequate compliance with instructions (duly annotated by the experimenter while observing performance) or blindly by the data extraction algorithm. Examples of automatically excluded and included recordings are shown in Supplementary Figure 1. We report data from 50 recordings obtained from 70 participants (30 individual recordings and 20 dyads, 37 female, mean age = 35.12 ± 13.41 years). Note that the relationship between “recording” and “participant” is not one to one: self recordings (i.e., Conditions ABC) involved one participants and pair recordings (i.e., Conditions DEF) involved two. For testing the hypotheses, the unit of analysis were the trials (3 trials per recording per condition) and determined the degrees of freedom in each test. In the interest of clarity, we also report the number of trials separately in each case.

**Figure 2.**
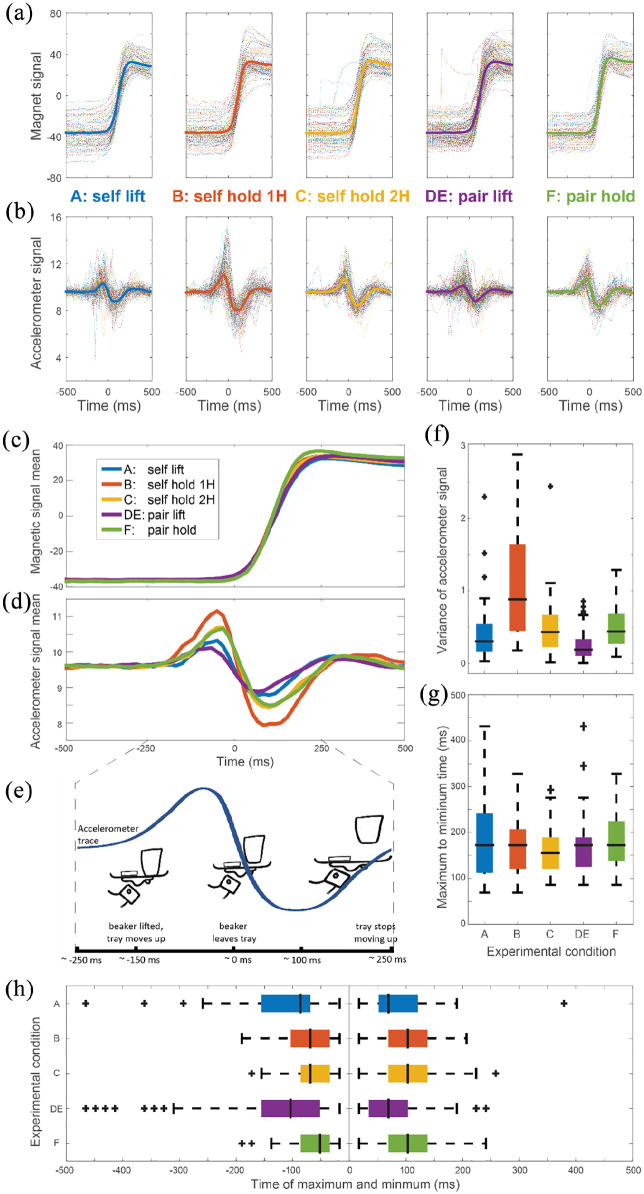
Tray movement analyses: (a) Magnetic signal in the axis perpendicular to the ground. Dotted lines represent individual trials. Thick lines depict the mean. (b) Same as A but for the accelerometer signal. (c) Superimposed average magnetic signal in the axis perpendicular to the ground across conditions. (d) Same as C but for the accelerometer signal. (e) Schematic representation of movement dynamics. (f) Accelerometer signal variance distributions for all the trial traces (whose mean is depicted in d). (g) Distributions of elapsed time between the positive (before 0 ms) and negative (after 0 ms) peaks of the acceleration traces. (h) Distributions of peak times. Left panel depicts maxima before 0 ms. Right panel depicts minima after 0 ms.

All analyses were performed on the vertical axis coordinate, and time locked to the time of beaker departure from the tray. To identify the moment of the beaker’s departure, the magnetic traces were analysed as follows: each recording was detrended (forced a zero mean) making the zero crossings the instantaneous moments of rapid change in magnetic signal (see Supplementary Figure 1 for two example recordings), that is, the ballistic movement of removing the beaker. After the detection and alignment of trials, time = zero was defined as the moment when the magnetic trace started rising (Figure 2c), and accelerometer signals were aligned accordingly (Figure 2d). Given that Conditions D and E were symmetrical (one of the members of the dyad lifted the beaker), they were lumped together and considered one condition we will call DE (pair-lift).

Variance of the acceleration was calculated for the time window from -500 to +500 ms (Figure 2f). To study the response times, we compared the peak and trough of the acceleration signal (Figure 2d). To study the time course of the responses, we compared the timings of the positive and negative deflections of the acceleration signal. For this, the time of the maximum peak was obtained from the window between -500 and 0 ms; and the time of the minimum trough was obtained from the window between 0 and +500 ms (Figure 2h). While the variance could be calculated for all the trials, positive and negative deflections were only visible in a substantial subset of the trials.

All data distribution quantification graphs presented are boxplots: black lines depict the median, filled coloured boxes depict first and third quartile, error bars (whiskers) depict ± 2.7 standard deviations and black crosses depict outliers.

All *p*-values declared for pairwise comparisons of distributions are derived from two-tailed Wilcoxon rank sum tests (equivalent to Mann–Whitney U tests) with an alpha level = 0.05, and adjusted for multiple comparisons with the Bonferroni method. The Kruskal–Wallis test was used to compare if the movement duration differed across groups. These tests were chosen because they are non-parametric and therefore more appropriate for data distribution that deviate from normality (as can be seen from the boxplots).

## Results

To investigate the tray-holding behaviour, we quantified tray movement across the experimental conditions. All trial traces were aligned to zero, defined as the moment of beaker departure from the tray (see mean traces by condition in Figure 2a and b, raw trial traces examples in Supplementary Figure 1). We used the acceleration signal from the smartphone on the tray as a proxy to analyse the stability of the tray while performing the task of removing the beaker across conditions. Therefore, we tested our hypotheses by comparing the timings and variability of the acceleration signals across conditions.

The accelerometer trace (Figure 2b and d) reflects the movement of the tray when the beaker was lifted. Initially, the beaker stays in contact with the tray as the upwards force applied by the person holding the tray remains constant (~-250 ms). The beaker leaves the tray when the tray holder starts to compensate for the change in the weight of the tray (~0 ms), until around ~500 ms the tray stops moving (Figure 2e).

First, to validate our methodology, we tested whether we could reproduce the well-established result (Hugon et al., 1982; Pezzulo et al., 2017) that self-coordination produces more stable movements than inter-individual coordination, that is, self-lifting (Condition A) is more stable than another person lifting (Condition B). To this end, we compared the variance of the accelerometer signal (Figure 2f) between conditions where the tray holders lifted the beaker (A for individuals, DE for dyads), and the conditions where lifting was performed by the experimenter (B for individuals, F for dyads). Both individual and dyad comparisons showed higher variance of the signal (i.e., less stability of the tray) when the experimenter lifted the beaker (Wilcoxon test: *P_A-B_* = 1.27e-13, z-score = -7.62, *N* of trials = [Condition A: 90, Condition B: 90]; *P_DE_*_-__F_ = 3.03e-09, z-score = -6.19, *N* of trials = [Condition DE: 120, Condition F: 60]).

Next, to test Hypothesis 1, we examined if there was less perturbation when the tray was held with two hands rather than one. We asked if individual participants held the tray more stably with two hands (Condition C) or one (Condition B) when the experimenter lifted the beaker. We confirmed that, when an individual held the tray with two hands there was less variability in the acceleration traces (Figure 2f, Wilcoxon: *P_B-C_* = 7.66e-09, z-score = -6.04, *N* of trials = [B: 90, C: 90]).

Having established that two hands from the same individual are more stable than one, we next tested Hypothesis 2. We asked whether two hands from different individuals (Condition F) were more stable than one hand from one individual (Condition B). Indeed, the results confirmed that they were (Wilcoxon: *P_B_*_-__F_ = 6.05e-07, *z*-score = 5.29, *N* of trials = [*B*: 90, *F*: 60]), meaning that two hands were more stable than one hand, regardless of whether the two hands belonged to one or two individuals. We also compared the variance for Condition DE (pair lift) with Condition A (self lift). Condition DE was slightly more stable than Condition A (Wilcoxon: *P_B_*_-__F_ = 0.0035, *z*-score = 3.45, *N* of trials = [*DE*: 120, *A*: 90]) This confirms that two hands are more stable than one, even when the two hands belong to different people.

Interestingly, when comparing conditions in which the two hands doing the holding were from the same individual (*C* self-hold 2 H) or from a dyad (*F* pair hold), we found no difference in the tray stability (Wilcoxon: *P_C_*_-_*F* = 0.61, *z*-score = -0.50, *N* of trials = [*C*: 90, *F*: 60]). This suggests that coordination between two individuals, in this task, was not worse (nor better) than self-coordination.

We then asked whether response times were different across conditions. Timings were quantified in terms of (1) how long the response event took, defined as the time elapsed between the maximum peak before beaker departure from the tray (0 ms) and the minimum peak afterwards (Figure 2g) and (2) how early the response onset started, defined as the time of the maximum peak relative to beaker departure from tray. We found no difference in the length of the response (Kruskal–Wallis: *p* = .39, chi-square = 4.15, *N* of trials = [*A*: 52, *B*: 69, *C*: 68, *DE*: 67, *F*: 43], degrees of freedom = 4), but we did see differences in the timing of response onset.

Our next prediction (hypothesis 3) stated that the response to the perturbation would be earlier when the beaker was lifted by someone holding the tray. Indeed, when participants lifted the beaker themselves, responses onset was earlier than when the experimenter did the lifting both for individuals (Wilcoxon: *P_A-B_* = 0.0025, *z*-score = -3.34, *N* of trials = [*A*: 69, *B*: 73]; *A_median_* = -86 ms, *B_median_* = -69 ms) and dyads (*P_DE_*_-__F_ = 4.57e-05, *z*-score = -4.33, *N* of trials = [*DE*: 93, *F*: 45]; *DE_median_* = -103 ms, *F_median_* = -52 ms) conditions (Figure 2h).

Finally, we examined whether there was a difference in response time when the tray was held by one or two people, themselves lifting the beaker (hypothesis 4, Figure 2h, A self lift and DE pair lift). Notably, we did not find a difference in response time in between these conditions (Wilcoxon: *P_A-DE_* = 0.43, *z*-score = 0.78, *N* of trials = [*A*: 69, *DE*: 93]).

## Discussion

In an interactive experiment conducted in the course of a public, science-engagement event at Tate Modern Museum in London, we replicate the earlier finding that there is less disruption in the stability of a tray when the person who lifts a beaker off the tray is also the one holding the tray. We also showed that there is less disruption when the tray is held by two hands. These results were expected. More surprising were the novel results indicating the degree of coordination when two people were holding the tray. There was less disruption in the stability of the tray even when the two hands holding it belonged to different people. Furthermore, the stability of the tray in this condition was not detectably worse than when the two hands belonged to one person. Similar conclusions were drawn when we looked at the timing of the compensatory response by the tray holder to the lifting of the beaker. This response onset was earlier when the beaker was lifted by the person who was holding the tray. Again, this was expected since the person initiating the action of lifting the glass off the tray could predict its timing and prepare the response of the hand holding the tray. More surprising was the finding that the response occurred equally early when a second person was holding the tray. This second person was not initiating the action, so how could they have anticipated the lifting of the beaker?

The most likely answer is that the haptic coupling established through the rigid tray provided a signal that was transmitted from the active, lifting holder to the passive holder. We have no direct evidence from our study concerning the nature of this signal. To investigate this idea, we would need to measure muscle activity and also have precise measures of the exact positions of the hands. This was not possible given the constraints of performing the experiment during an open day at an art gallery. However, we can speculate on the basis of previous work on the active lifting of loads.

Whenever we move, we make anticipatory postural adjustments. These adjustments are necessary, for example, to take account of any change in our centre of gravity that the movement might cause (Belenkiy et al., 1967). Such postural adjustments occur during active lifting (Hugon et al., 1982). In this experiment, participants had a weight resting on one arm, and in an active condition, removed this weight with their contralateral hand. During the performance of this task, electromyographic activity (EMG) of biceps and triceps muscles of both arms was recorded. An anticipatory decrease in muscle activity was observed in the load bearing arm, occurring ~20 ms *before* the lift. In a passive condition, when the experiment removed the load, this change in muscle activity did not occur until ~65 ms *after* the lift. The authors suggested that, in the active lifting condition, this anticipatory postural adjustment in the load bearing arm resulted from a feedforward control of the flexors of this arm which served to minimise the effects of the disturbance caused by the other arm’s lifting movement.

In our study, the load bearing arm is the arm holding the tray. So, when that person’s other arm lifts the beaker, we would expect to see preparatory muscular activity in the holding arm. We speculate that this preparatory activity in the load bearing arm could act as a signal, transmitted through the rigid platform of the tray, that would elicit preparatory activity in the arm of the other person holding the tray. The importance of such haptic signals in enabling coordination between people has implications for the design of systems involving joint action, whether between people or between people and artificial agents such as health-care providing robots and self-driving cars.

Romero and colleagues (2015) studied interpersonal hand coordination and found greater synergy for interpersonal than intrapersonal interaction between arms. In other words, when individuals work together, their movements can become temporarily organised to form a single synergistic two-person system. Such a system requires *reciprocal compensation*, that is mutual adaptation between the partners in the timing of their movements (see, e.g., Konvalinka et al., 2010). Our results suggest that haptic connectivity may play a crucial role in enabling recurrent compensation.

Conducting our study in a public event had many advantages. Within the space of 3 days, we interacted with many curious and enthusiastic participants who generously offered their time and opinion to our project. Running the study in a bustling public space very similar to an actual party where one might be offered drinks off a tray provided a strong ecological validity. The robustness of the results testified to the reliability of our methodology and experimental design. We took advantage of two affordances of typically available smart phones, namely the gyroscopic movement sensor and the magnetic field sensor to construct a simple system to measure the dynamics of social interaction. We have provided all of our experimental code and how-to tutorial documentation as Supplementary Material to this paper and we would be delighted to see other laboratories using them for their projects.

## Supplemental Material

sj-docx-1-qjp-10.1177_17470218211009082 – Supplemental material for Helping the waiter to hold his tray: Rigid haptic linkage promotes inter-personal motor coordination Click here for additional data file. Supplemental material, sj-docx-1-qjp-10.1177_17470218211009082 for Helping the waiter to hold his tray: Rigid haptic linkage promotes inter-personal motor coordination by Dardo N Ferreiro, Chris D Frith and Bahador Bahrami in Quarterly Journal of Experimental Psychology
