# Supplementary material for: Helping the waiter to hold his tray: Rigid haptic linkage promotes inter-personal motor coordination
Source: Q J Exp Psychol (Hove). 2021 Apr 12;74(10):1784–90. doi: 10.1177/17470218211009082 (PMC8392761; doi:10.1177/17470218211009082)
Supplement: sj-docx-1-qjp-10.1177_17470218211009082 – Supplemental material for Helping the waiter to hold his tray: Rigid haptic linkage promotes inter-personal motor coordination [file sj-docx-1-qjp-10.1177_17470218211009082.docx]

**Supplementary Material for:**

**Helping the waiter to hold his tray: rigid haptic linkage promotes inter-personal motor coordination**

Dardo N. Ferreiro, Chris D. Frith, Bahador Bahrami


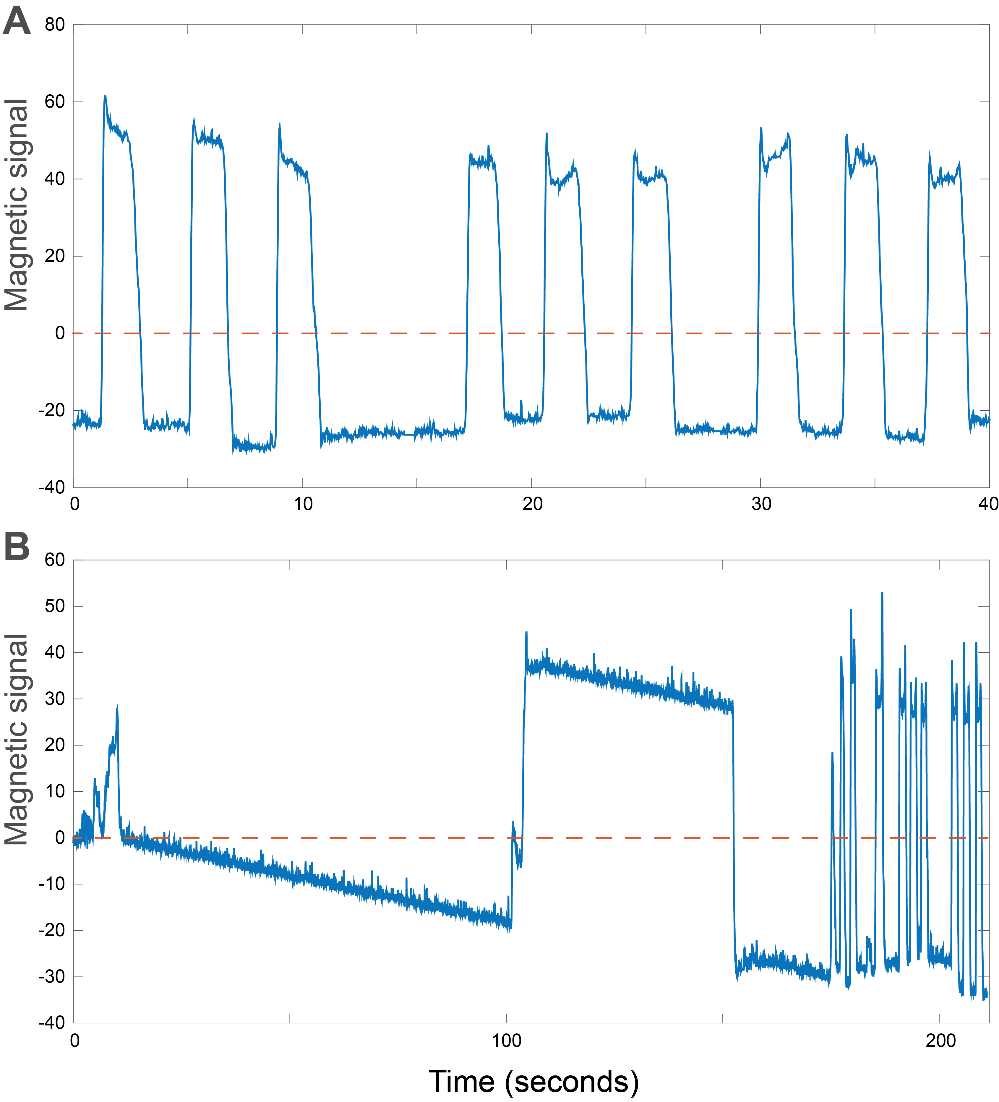


Supplementary Figure 1. Examples of magnetic signals in the Z axis from two different recordings. (A) A recording that was included in the analyzed data. The three trials in each of the three conditions are clearly visible. (B) A recording that excluded from analyses. Blue continuous line depicts the signal and red dotted lines depict the zero. Original signals were detrended (forced a zero mean) and included in further analyses only if they presented 18 zero-crossings (corresponding to 9 events of lifting the glass from the tray, as participants were instructed to do in every recording).
